# Hyperglycemia-Induced Dysregulated Fusion Intermediates in Insulin-Secreting Cells Visualized by Super-Resolution Microscopy

**DOI:** 10.3389/fcell.2021.650167

**Published:** 2021-04-15

**Authors:** Guoyi Yang, Liuju Li, Yanmei Liu, Kuo Liang, Lisi Wei, Liangyi Chen

**Affiliations:** ^1^State Key Laboratory of Membrane Biology, Beijing Key Laboratory of Cardiometabolic Molecular Medicine, Institute of Molecular Medicine, School of Future Technology, Peking University, Beijing, China; ^2^Institute for Brain Research and Rehabilitation, Key Laboratory of Brain, Cognition and Education Science, South China Normal University, Guangzhou, China; ^3^Department of General Surgery, Xuanwu Hospital, Capital Medical University, Beijing, China; ^4^PKU-IDG/McGovern Institute for Brain Research, Beijing, China; ^5^Beijing Academy of Artificial Intelligence, Beijing, China; ^6^Shenzhen Bay Laboratory, Shenzhen, China

**Keywords:** fusion pore, exocytosis, SNARE, secretory vesicle, insulin, glucotoxicity, structured illumination microscopy

## Abstract

Impaired insulin release is a hallmark of type 2 diabetes and is closely related to chronically elevated glucose concentrations, known as “glucotoxicity.” However, the molecular mechanisms by which glucotoxicity impairs insulin secretion remain poorly understood. In addition to known kiss-and-run and kiss-and-stay fusion events in INS-1 cells, ultrafast Hessian structured illumination microscopy (Hessian SIM) enables full fusion to be categorized according to the newly identified structures, such as ring fusion (those with enlarged pores) or dot fusion (those without apparent pores). In addition, we identified four fusion intermediates during insulin exocytosis: initial pore opening, vesicle collapse, enlarged pore formation, and final pore dilation. Long-term incubation in supraphysiological doses of glucose reduced exocytosis in general and increased the occurrence of kiss-and-run events at the expense of reduced full fusion. In addition, hyperglycemia delayed pore opening, vesicle collapse, and enlarged pore formation in full fusion events. It also reduced the size of apparently enlarged pores, all of which contributed to the compromised insulin secretion. These phenotypes were mostly due to the hyperglycemia-induced reduction in syntaxin-1A (Stx-1A) and SNAP-25 protein, since they could be recapitulated by the knockdown of endogenous Stx-1A and SNAP-25. These findings suggest essential roles for the vesicle fusion type and intermediates in regulating insulin secretion from pancreatic beta cells in normal and disease conditions.

## Introduction

Insulin is a physiological hormone released from pancreatic beta cells and plays a vital role in regulating blood glucose levels. In mammalian cells, an increase in glucose blocks ATP-dependent potassium channels, leading to membrane depolarization and the influx of calcium that triggers insulin granule exocytosis (Flatt et al., 1980). Glucose-stimulated insulin secretion (GSIS) is compromised in diabetes, which, in part, leads to the long-term hyperglycemia condition first proposed by Unger et al. in the 1980s as “glucotoxicity” (Unger and Grundy, 1985; Ostenson and Efendic, 2007; Bensellam et al., 2012). The molecular mechanisms underlying glucotoxicity, however, have not been fully explored. In addition to enhanced ER stress (Lemaire and Schuit, 2012), cell apoptosis (Tomita, 2016), altered Ca^2+^ signaling (Klec et al., 2019), and reduced insulin secretion ability are associated with decreased SNARE proteins (Gaisano et al., 2002; Zhang et al., 2002). Interestingly, insulin granules are released from fusion pore intermediate structures, as demonstrated indirectly by optical imaging (Takahashi et al., 2002) and directly by electrophysiological methods (MacDonald et al., 2006), which could help more selectively release of neurotransmitters over insulin from the granules. It has been suggested that *SOX4*, which is overexpressed in diabetes, may reduce insulin secretion by impairing fusion pore expansion (Collins et al., 2016). These data indicate a possible link between fusion intermediate dysregulation and defective insulin secretion in diabetes, although a systematic investigation is needed.

Fusion pores were first detected by the electrochemistry method (Chow et al., 1992). Hormone release from granules oxidized by a carbon fiber electrode (CFE) was detected as an electric current with “foot signals,” which are thought to arise from the restricted diffusion of molecules by narrow pores (Wightman et al., 1991; Zhou et al., 1996). In combination with membrane capacitance recording (Albillos et al., 1997), the opening and reversible flickering of a small pore in some fusion events was established as “kiss-and-run” (MacDonald et al., 2006; Hanna et al., 2009). Additionally, methods based on real-time fluorescence imaging have also been used to detect the regulation of vesicle secretion and to probe the spatial profiles of vesicle exocytosis (Anantharam et al., 2010; Zhao et al., 2013; Bendahmane et al., 2018). However, with a limited spatiotemporal resolution, these imaging methods mainly indirectly classify different types of exocytosis based on fluorescently tagged proteins’ kinetics after fusion (Guček et al., 2019). Recently, we developed ultrasensitive Hessian structured light illumination microscopy (Hessian-SIM) (Huang et al., 2018). Meanwhile, we labeled VAMP2 (vesicle-associated membrane protein-2/synaptobrevin) with the pH-sensitive variant of GFP (pHluorin) to visualize single exocytotic vesicles. The pHluroin attached to the lumenal side of VAMP2 is quenched before exocytosis. During exocytosis, the vesicle fuses with the plasma membrane and exposes its lumen. The dequenching of luminal pH from 5.5 to 7.3 leads to an increase in the pHluorin fluorescence, which marks vesicle fusion (Miesenböck et al., 1998). Thus, this technique enables enlarged fusion pores and other millisecond fusion intermediates labeled with VAMP2-pHluorin to be observed in live INS-1 cells.

In the current study, Hessian SIM imaging in INS-1 cells cultured in different glucose concentrations demonstrated that long-term hyperglycemia reduced secretion events and switched the vesicle fusion mode from full fusion to kiss-and-run fusion. Besides, hyperglycemia also delayed fusion pore opening and vesicle collapse, reduced the size of enlarged fusion pores, and hindered full fusion pore expansion. Overall, we demonstrate possible mechanisms underlying insulin secretion that are compromised by different levels of sustained glucose exposure.

## Results

### INS-1 Cell Exocytosis Mainly Exhibited Full-Fusion and Kiss-and-Run Fusion Modes

**FIGURE 1:**
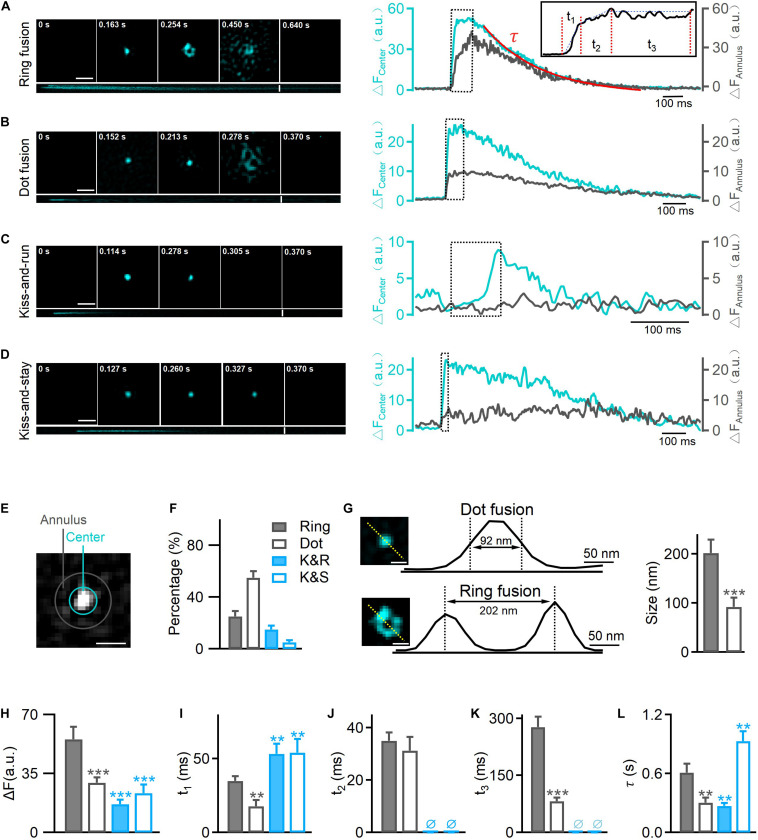
Hessian-TIRF-SIM reveals four types of exocytosis. INS-1 cells were transfected with VAMP2-pHluorin and stimulated with 70 mM KCl and 20 mM glucose. **(A)** Left: representative SIM images showing ring fusion events. Montages (upper) and kymographs (lower) of a ring fusion event in vesicles labeled with VAMP2-pHluorin (cyan). Scale bar, 300 nm; frame rate, 291 Hz [with the “rolling” procedure (Huang et al., 2018)]. Right: changes in fluorescence intensities of annulus (gray) and center (cyan) regions of the fusion event shown in **(E)**. Inset: a high magnification of the trace. Durations **t_1_**, **t_2_**, and **t_3_** and the decay constant **τ** (fitted with a single exponential function) describe the durations of different intermediates: the initiation of fusion pore opening, collapse of the vesicle with the plasma membrane, formation of enlarged pores, and final dilation, respectively. **(B–D)** The montages, kymographs, and time-dependent fluorescence traces of a representative dot fusion **(B)**, a kiss-and-run event **(C)**, or a kiss-and-stay event **(D)**. Notably, the fluorescence intensities at the annulus region surrounding the exocytotic site exhibited either no change under kiss-and-run fusion (gray, **C**, right) or a small and delayed increase under kiss-and-stay fusion (gray, **D**, right). **(E)** The annulus (gray) represents the diffusion area between the inner (10 pixels in diameter) and outer (16 pixels in diameter) circles; the center (cyan) represents the inner circle of the fusion pore area. Scale bar, 200 nm. **(F)** Percentages of ring fusion, dot fusion, kiss-and-run exocytosis, and kiss-and-stay exocytosis evoked by 70 mM KCl and 20 mM glucose (*n* = 150 fusion events from 10 cells). **(G)** The fluorescence intensity profiles for the dotted lines across the enlarged pore (lower) and fluorescence puncta (upper). Diameters were measured as the full width at half maximum (FWHM) of the fluorescence valley (lower, pore size) and peak (upper, puncta size), which gave sizes of 202 ± 27 nm (*n* = 35 fusion events) and 92 ± 19 nm (*n* = 40 fusion events) for the pore and puncta, respectively. Scale bar, 100 nm. **(H–L)** Averaged calculations of **t_1_**, **t_2_**, **t_3_**, **τ**, and the amplitude change of different types of exocytosis (*n* = 300 fusion events from 6 cells). *p < 0.05, **p < 0.01, ***p < 0.001.

Insulin granule exocytosis was evoked by a depolarizing solution containing high K^+^ (70 mM KCl and 20 mM glucose), and was continuously recorded in real-time for 10 min at a frame rate of 291 Hz using the TIRF-SIM microscope. The fusion events were characterized by a transient fluorescence increase at the center of the VAMP2-pHluorin molecule intensity, followed by a decay to baseline (Figures 1A–D).

Regarding the pHluorin-labeled events, we observed four categories of fusion modes according to their fluorescence intensity profile and diffusion pattern: ring fusion (full fusion with a “ring” structure, Figure 1A), dot fusion (full fusion with puncta, Figure 1B), kiss-and-run (K&R, Figure 1C) and kiss-and-stay (K&S, Figure 1D) according to their fluorescence intensity profile and diffusion pattern. In full fusion events (ring and dot fusion), corresponding to spreading or discharge, a robust fluorescence increase occurred both at the center and in the annular area of VAMP2-pHluorin molecules (Figures 1A,B,E), indicating the full collapse of a vesicle’s one-time diffusion to the plasma membrane and a dilated fusion pore. In contrast, confined events (kiss-and-run and kiss-and-stay) showed a brightening of the central intensity but no or a limited fluorescence increase in the annular area (Figures 1C–E), representing transient opening and reclosure of a restricted fusion pore that limits vesicle collapse.

Markedly, 80% of the total vesicle exocytotic events were full fusion (Figure 1F), including ring fusion (25%, FWHM ∼202 ± 27 nm in diameter, Figures 1F,G) and dot fusion (55%, FWHM ∼92 ± 19 nm in diameter, Figures 1F,G), when depolarizing stimulation using high K^+^ solution were applied to INS-1 cells. The percentages of kiss-and-run and kiss-and-stay events were 15 and 5%, respectively (Figure 1F). Collectively, the data indicate that after stimulation by membrane depolarization, full fusion and Kiss-and-run fusion are the dominant release modes in INS-1 cell exocytosis.

Next, we characterized the kinetics of single fusion events. A previous study showed that for the ring and dot fusions, with an improved frame rate of 291 Hz [via a “rolling” SIM reconstruction (Huang et al., 2018), four kinetically distinct steps could be dissected (Huang et al., 2018)]: (1) an initial rapid increase in the fluorescence intensity (fast rise, duration **t_1_**), which appeared to represent the initial pore opening when vesicular H^+^ efflux occurred; (2) a slower increase (slow rise, duration **t_2_**) due to the movement of the fusing vesicle toward the plasma membrane; and (3) a stage in which the fluorescence remained elevated (plateau phase, duration **t_3_**) and during which the enlarged fusion pore formed; and (4) the final return of fluorescence to the baseline value (decay phase, the time constant **τ**), which refers to the final dilation of the vesicle (Figure 1A, higher magnification inset, Figure 5). Meanwhile, for kiss-and-run and kiss-and-stay events, the fusion of single vesicles exhibited only the fast rise phase **t_1_** and the decay phase (time constant **τ**); the slow-rise and plateau phases were absent (Figures 1C,D). The rising phase probably represents fusion pore opening, and the decay phase may depict the vesicle resealed and reacidification of the vesicle lumen at the kiss-and-run events (Figure 5).

Further, we found that the average **t_1_**, **t_3_**, and **τ** values for ring fusion were 1.5–3.5-fold longer than those for dot fusion events. Given the rate of change in fluorescence was the same in the two populations according to a previous study (Huang et al., 2018), more time is needed for the dequenching or diffusion of VAMP2 in vesicles undergoing ring events. Simultaneously, the mean peak intensity of ring events was 1.5-fold higher than that of dot events, suggesting that ring events involve vesicles with larger membrane surfaces (Figure 1H). Among the confined events, the rise times are similar between kiss-and-run (∼60 ms) and kiss-and-stay (∼64 ms) events (Figure 1I), but were markedly slower than full fusion events, in agreement with smaller fusion pores. In contrast, the vesicle collapse times of full fusion were quite similar (Figure 1J). In addition, ring events remained in the enlarged fusion pore intermediate phase (**t_3_**) for more extended periods (Figure 1K). Finally, the decay time (**τ**) of kiss-and-stay events was 3.5-fold longer than that of kiss-and-run events, suggesting very slow diffusion of vesicular proteins on the plasma membrane (Figure 1L).

### Sustained Exposure of INS-1 Cells to High Glucose Concentrations Decreased the Full Fusion Frequency and Evoked More Liss-and-Run Events

To better explore the mechanism by which chronic hyperglycemia impairs GSIS, we established a glucose toxicity model in INS-1 cells. Briefly, glucotoxicity conditions were created by exposing INS-1 cells to RPMI 1640 full culture medium containing 20 or 30 mM glucose for 120 h. The cells were then used to detect cell function under GSIS conditions. Using ELISA experiments to detect insulin secretion, we showed that (1) for INS-1 cells cultured in the presence of basal (5 mM) glucose, stimulation with 16.7 mM glucose produced a threefold enhancement of insulin secretion; and (2) with increasing concentrations of glucose in the medium, the secretory response to 16.7 mM glucose was reduced by 40–68% in cells cultured under high glucose (*p* < 0.01) (Supplementary Figure 1).

**FIGURE 2:**
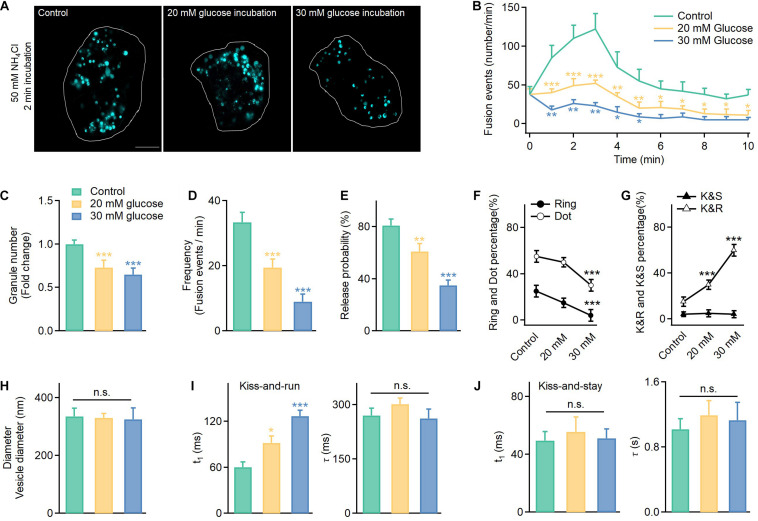
In addition to reduced exocytosis, hyperglycemia leads to more kiss-and-run events at the expense of full fusion events in INS-1 cells. **(A)** Vesicles labeled with VAMP2-pHluorin after NH_4_Cl incubation (50 mM, 2 min) observed in control cells cultured in normal glucose (11 mM) or supraphysiological glucose (20 and 30 mM) for 120 h. Scale bar: 3 μm. **(B)** Time-dependent exocytosis triggered by 20 mM glucose and 70 mM KCl in INS-1 cells cultured under different glucose concentrations. **(C–E)** Normalized total vesicle numbers (**C**, Control: *n* = 180 vesicles; 20 mM: *n* = 186 vesicles; 30 mM: *n* = 182 vesicles), release frequency **(D)**, and release probability (normalized to the total vesicle number, **E**). **(F,G)** The percentage of ring fusion, dot fusion **(F)**, kiss-and-run, and kiss-and-stay events **(G)** under different glucose concentrations. **(H)** Vesicle diameters in INS-1 cells cultured under different glucose concentrations (control: *n* = 180 vesicles; 20 mM: *n* = 186 vesicles; 30 mM: *n* = 182 vesicles). **(I,J)** The **t_1_** and τ of kiss-and-run (**I**, *n* = 80) and kiss-and-stay events (**J**, *n* = 60 fusion events) under different glucose concentrations. *p < 0.05, **p < 0.01, ***p < 0.001.

Next, to determine whether high glucose treatment inhibits vesicle synthesis, we counted the number of vesicles labeled with VAMP2-pHluorin after NH_4_Cl incubation (50 mM, 2 min) under control (11 mM glucose) and hyperglycemic conditions (high glucose: 20 and 30 mM) (Figure 2A). Although diameters of granules in cells cultured in 20 and 30 mM glucose remained unchanged (Figure 2H and Supplementary Figure 3), hyperglycemia significantly reduced the number of secretory vesicles (Figure 2C). This result agreed with the reduced insulin granules immunofluorescently labeled in INS-1 cells cultured under elevated glucose concentrations (Supplementary Figure 2). Meanwhile, the hyperglycemic conditions severely reduced the fusion frequency (Figures 2B,D) and the probability of vesicle release (Figure 2E), confirming the reduced exocytosis in cells after long-term incubation in high glucose concentrations (Supplementary Figure 1).

Thus, we examined the fusion mode. Surprisingly, long-term exposure to high glucose concentrations reduced the full fusion percentage (Figure 2F, ring fusion: ∼25–5%; dot fusion: ∼55–30%) and triggered more kiss-and-run events (∼15–60%), while the percentage of kiss-and-stay events remained unchanged (∼5%) (Figure 2G). Regarding whether high glucose treatment altered the kinetics of the confined events, the results showed a slower rise time for kiss-and-run events, which reflected a longer brightening time for the pHluorin molecules and a smaller fusion pore (Figure 2I). In contrast, kiss-and-stay events showed more tolerance to glucotoxicity since the rise and decay times showed no significant differences compared to the events under control glucose concentrations (Figure 2J).

### Restricted Fusion Pore, Delayed Opening, Vesicle Collapse, and Enlarged Pore Formation of Full Fusion Events Under Long-Term Hyperglycemia Conditions

**FIGURE 3:**
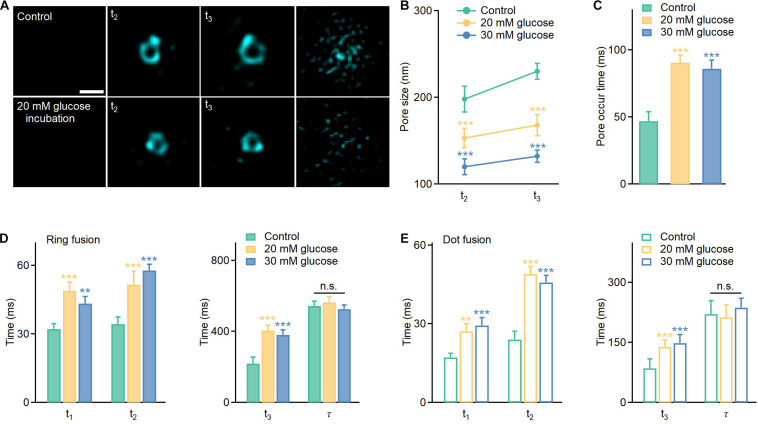
Hyperglycemia delays the opening of the fusion pore, vesicle collapse, and enlarged pore formation in full fusion events and reduces the size of apparently enlarged pores. **(A)** Representative images of enlarged pores at the **t_2_** and **t_3_** stages under different conditions. Scale bar: 200 nm. **(B,C)** Average pore sizes and pore occur times under different conditions (Control: *n* = 80 fusion events from 25 cells; 20 mM glucose: *n* = 120 fusion events from 20 cells; 30 mM glucose: *n* = 90 fusion events from 20 cells). **(D,E)** Effects of long-term hyperglycemia on fusion intermediates of ring fusion and dot fusion (ring fusion: *n* = 50 fusion events from 10 cells; dot fusion: *n* = 60 fusion events from 8 cells). *p < 0.05, **p < 0.01, ***p < 0.001.

Next, we investigated spatiotemporal kinetics of full fusion events revealed by the Hessian SIM, and compared the fusion pore sizes at **t_2_** (vesicle collapse) and **t_3_** (enlarged pore formation) under normal (11 mM glucose) and long-term hyperglycemia (20 mM and 30 mM glucose, 120 h) conditions. The results showed that glucotoxicity reduced the fusion pore size (Figures 3A,B), from 198 ± 15 nm (Control, *n* = 30) to 153 ± 11 nm (20 mM, *n* = 25) and 120 ± 9 nm (30 mM, *n* = 35) at the **t_2_** stage, and from 230 ± 12 nm (Control, *n* = 30) to 168 ± 12 nm (20 mM, *n* = 30) and 132 ± 10 nm (30 mM, *n* = 20) at the **t_3_** stage. Correspondingly, long-term exposure to glucose also delayed ring fusions. A longer period of time was needed to observe the first appearance of pore structures (Figure 3C), along with delayed initial fusion pore opening (Figure 3D, **t_1_**), vesicle collapse, and extended large pore formation (Figure 3D, **t_2_**, **t_3_**). The final dilation and diffusion of the vesicular membrane, however, was not affected. Similarly, **t_1_**, **t_2_**, and **t_3_** were all slowed in dot fusion events from cells been treated with high glucose for 120 h (Figure 3E).

### Hyperglycemia-Induced Reduction in SNARE Protein Expressions Contributes to the Altered Fusion Dynamics

**FIGURE 4:**
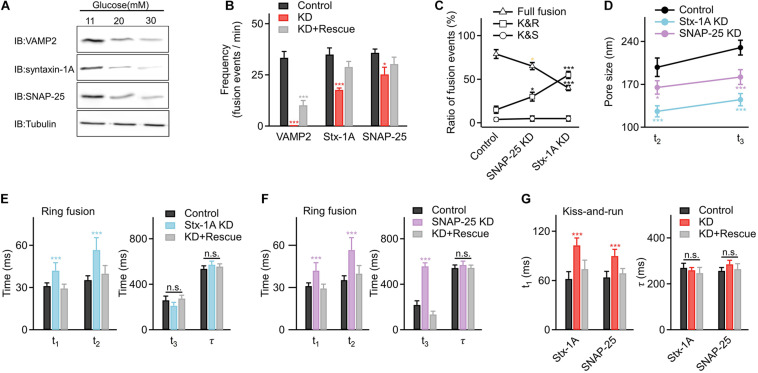
Hyperglycemia alters fusion kinetics by decreasing the expression of the core SNARE complex in INS-1 cells. **(A)** Representative examples of reduced expression of SNARE proteins in INS-1 cells cultured at elevated glucose concentrations. Tubulin was used as a control (four repeats in each condition). **(B)** Fusion frequencies in control INS-1 cells (*n* = 25 cells); cells with knockdown (KD) of VAMP2 (*n* = 10 cells), syntaxin-1A (Stx-1A) (*n* = 16 cells), or SNAP-25 (*n* = 18 cells), and KD cells overexpressing designated SNARE proteins as the rescue (KD+ Rescue) (VAMP2: *n* = 10 cells; syntaxin-1A: *n* = 16 cells; SNAP-25: *n* = 18 cells). **(C)** Percentage of full fusion, kiss-and-run and kiss-and-stay events upon KD of SNARE proteins (Control: *n* = 90 fusion events from 7 cells; Stx-1A KD: *n* = 60 fusion events from 8 cells; SNAP-25 KD: *n* = 85 fusion events from 9 cells). **(D)** Average sizes of enlarged fusion pores during the **t2** and **t3** stages upon KD of SNARE proteins (Control: *n* = 35 fusion events from 6 cells; Stx-1A KD: *n* = 25 fusion events from 10 cells; SNAP-25 KD: *n* = 30 fusion events from 10 cells). **(E,F)** Fusion pore intermediates of ring fusion upon KD of syntaxin-1A **(E)**, SNAP-25 **(F)** (Control: *n* = 35 fusion events from 6 cells; Stx-1A KD: *n* = 25 fusion events from 10 cells; SNAP-25 KD: *n* = 30 fusion events from 10 cells) and SNARE proteins KD+ Rescue (Stx-1A: *n* = 30 fusion events from 7 cells; SNAP-25: *n* = 26 fusion events from 10 cells). **(G)** Dynamics of kiss-and-run events in INS-1 cells with KD of syntaxin-1A, SNAP-25 (Control: *n* = 25 fusion events from 6 cells; Stx-1A KD: *n* = 20 fusion events from 7 cells; SNAP-25 KD: *n* = 30 fusion events from 7 cells) and SNARE proteins KD+ Rescue (Stx-1A: *n* = 25 fusion events from 5 cells; SNAP-25: *n* = 30 fusion events from 8 cells). *p < 0.05, **p < 0.01, ***p < 0.001.

To better explore the underlying mechanisms, we further determined whether glucotoxicity altered expressions of key regulators in vesicle fusion, SNARE proteins. Surprisingly, we found that core SNARE complex expression was significantly reduced at higher glucose concentrations (Figure 4A and Supplementary Figure 4). Correspondingly, knocking down syntaxin-1A and SNAP-25 in INS-1 cells decreased the fusion frequency (Figure 4B), triggered more kiss-and-run events (Figure 4C), and restricted the fusion pore size (Figure 4D), which were similar to the effects of hyperglycemia.

Next, we determined how the fusion pore dynamics changed when interfering with syntaxin-1A and SNAP-25. For ring fusion, decreased syntaxin-1A expression slowed early pore opening and inhibited vesicle collapse to the plasma membrane (Figure 4E), while decreased SNAP-25 expression slowed early pore opening, inhibited vesicle collapse, and hindered the enlarged pore formation process (Figure 4F). Similar results occurred in dot fusion (Supplementary Figures 5A,B). In addition, decreased SNARE expression also inhibited the initial pore opening of kiss-and-run events (Figure 4G) but had no significant effect on kiss-and-stay events (Supplementary Figure 5C), phenocopied the effects of long-term exposure to high glucose in INS-1 cells.

### Proposed Working Model: Long-Term Exposure to High Glucose Changed the Fusion Mode and Inhibited Fusion Pore Dynamics

**FIGURE 5:**
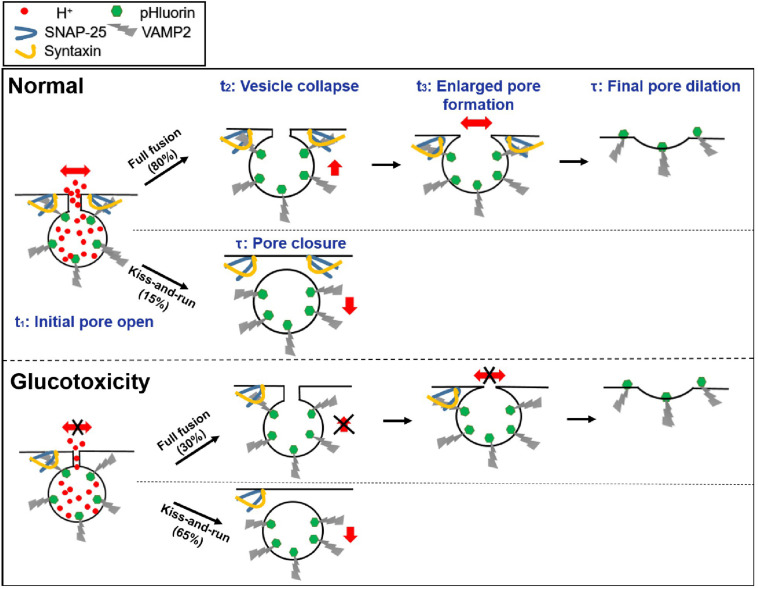
Proposed model of exocytosis under normal and glucotoxicity conditions.

Hessian SIM microscopy enabled the direct visualization of the insulin fusion process and led us to the following conclusions:

1. Glucotoxicity triggers more kiss-and-run events while decreasing full fusion events, which may reduce insulin secretion.
2. For full fusion events, glucotoxicity changes the fusion pore dynamics by restricting the fusion pore size, hindering fusion pore opening, delaying vesicle collapse, and inhibiting the pore expansion process. For kiss-and-run events, glucotoxicity only inhibits fusion pore opening (Figure 5).
3. Glucotoxicity regulates fusion pore dynamics by decreasing SNARE complexes, which may be the potential mechanism leading to defective insulin secretion in diabetes.

## Discussion

Here, we revealed that glucotoxicity impacts insulin secretion at three different levels. While the reduction in glucose-stimulated fusion events agrees, in general, with previous studies (Kawahito et al., 2009), the increase in the relative ratio of kiss-and-run fusion came at the cost of reduced full fusion. The relative occurrence of kiss-and-run in insulin-secreting cells varies widely, from completely absent (Ma et al., 2004) to the vast majority of events (Tsuboi and Rutter, 2003). Exogenously overexpressing VAMP2-pHlourin may perturb the intrinsic secretion properties of insulin granules. Fusion dynamics may differ upon different vesicular labeling strategies (Rutter et al., 2006; Caromile et al., 2010). However, both the numbers of insulin granules detected by the immunofluorescence microscope (Supplementary Figure 2) and the secretion detected by the ELISA experiments (Supplementary Figure 1) agreed nicely with experiments conducted with VAMP2-pHlourin (Figures 2A–D). Therefore, we believe that the exogenously expressed VAMP2-pHlourin does not significantly affect the exocytosis of insulin granules. By defining fusion events with no diffusion of VAMP2-pHluorin from the release sites as kiss-and-run events (Figure 1C), the percentage of these events increased from 15 to 60% in cells cultured with 30 mM glucose, suggesting an important role of insulin release regulated by the fusion mode.

By applying Hessian SIM, we identified enlarged pore formation in some fusion events, which led to the separation of full fusion into subcategories of ring fusion and dot fusion. These enlarged pores resembled similar structures observed under stimulated emission depletion (STED) microscopy (Shin et al., 2018). However, because of the intense phototoxicity and photobleaching associated with STED, long time-lapse live-cell imaging of hundreds of fusion events for statistical analysis was not possible previously. These enlarged pores are different in size from pores several nanometers in diameter detected either by electrochemical or electrophysiological methods (Alvarez de Toledo et al., 1993; MacDonald et al., 2006) and thus may not function to prioritize the release of small over large cargoes. However, these greater than ∼100 nm pores are similar to those in images captured with a rapid-freezing electron microscope more than 30 years ago (Ornberg and Reese, 1981). Hessian SIM enabled hundreds of events to be recognized in live INS-1 cells, thus further validating the general relevance of this phenomenon. Moreover, millisecond temporal resolution enabled intricate dynamics that last only ∼30 ms to be resolved, such as the initial abrupt increase in pHluorin intensity resembling vesicular H^+^ efflux via opened pores and the slow increase in pHluorin intensity resembling movement of the fusing vesicle toward the plasma membrane, which could not be separated by super-resolution microscopy, even when operating at the video frame rate. Having characterized these four intermediates, we were able to show that hyperglycemia inhibited the initial opening, delayed vesicle collapse, reduced enlarged pore sizes, and prolonged the pore duration (Figure 3). These data suggest possible delayed and reduced insulin release even in full fusion events, which represents the final layer of glucotoxic effects.

Chronic incubation of INS-1 cells or beta cells in high glucose for 72 h, 96 h, and 5-days’ have been tested previously (Unger and Grundy, 1985; Ostenson and Efendic, 2007; Bensellam et al., 2012). In our case, we employed a high-glucose incubation period of 120 h (5 days) to simulate similar clinical hyperglycemia conditions. Indeed, decreased expression of exocytotic SNARE proteins in pancreatic islets is a postulated mechanism behind impaired insulin release, demonstrated by significantly reduced mRNA and protein levels in both rodent models of type 2 diabetes (Nagamatsu et al., 1999; Gaisano et al., 2002; Zhang et al., 2002) and islets of type 2 diabetic patients (Ostenson and Efendic, 2007). We also showed that long-term hyperglycemia led to reduced SNAP-25 and syntaxin-1A expression (Figure 4A and Supplementary Figure 4), which may constitute the major factor for reshaping vesicle fusion dynamics. Interestingly, we found different effects when reducing endogenous syntaxin-1A compared to SNAP-25. Interfering with either syntaxin-1A or SNAP-25 slowed the initial pore opening and inhibited vesicle collapse to the plasma membrane (Figure 4E) while reducing only SNAP-25 prolonged the formation of enlarged pores (Figure 4F). Thus, different components of the SNARE complex may have different regulatory effects on the opening-to-expansion process of the fusion pores. Another possible explanation is that BoNT/C α51 depletes syntaxin-1A predominantly but may not affect syntaxin-3 and syntaxin-4, which mediate newcomer granule fusion (Zhu et al., 2013; Xie et al., 2015). These remaining syntaxins may be able to assist the expansion of the fusion pore.

We also noticed that the kiss-and-stay granules were not significantly affected by reducing SNARE protein levels (Figure 2J). Because the proportion of kiss-and-stay fusion to total fusion is around 5%, it may use only a small portion of all SNARE complexes in the INS-1 cell. Thus, decreased number of SNARE complex may have mild effects on this kind of fusion event.

Besides, from the perspective of fusion kinetics, we found that the initial pore opening (**t_1_**) and pore formation time (**t_3_**) in dot fusion are much faster than that in the large fusion pore ring structure (Figures 1I,K). This may indicate that the dot fusion may require fewer SNARE proteins and is more likely to be initiated than ring fusion. In contrast, large vesicles may require more SNARE complexes to maintain the pore opening and subsequent fusion (Weber et al., 1998; Shi et al., 2012; Bao et al., 2018).

In addition to the core SNARE proteins, many other accessory proteins interact with the SNARE complex (e.g., NSF/SNAPs, Rab family members, Sec1/Munc18, synaptotagmin, Munc13, and complexin) during the fusion process to jointly regulate the secretion process. For example, Munc18 is often considered to be the protein most closely related to the assembly of SNARE proteins (Rizo and Südhof, 2002), while synaptotagmin acts as a Ca^2+^ sensor for regulated secretion (Sudhof, 2004). Further work is needed to clarify the function of these proteins in fusion pore regulation.

Although this study was carried out in INS-1 cells, the glucotoxicity model may simulate the hyperglycemic environment in primary beta cells to some extent. Furthermore, the functions of the involved proteins are conserved in primary beta cells. Thus, the mechanisms found here may be extrapolated to defective insulin secretion in diabetes.

## Research Design and Methods

### Plasmid Expression

To study the fusion pore dynamics, INS-1 cells were transfected with VAMP2-pHluorin using Lipofectamine 2000 reagent (Thermo Fisher Scientific, 11668019) and plated onto polylysine-coated coverslips. The experiments were conducted after 16–24 h of culturing in an incubator at 37°C. For the knockdown and rescue experiments, cells were transfected with BoNT/C α51, BoNT/E, syntaxin1A-EGFP, and SNAP-25-EGFP.

### INS-1 Cell Culture and Preparation

The rat insulinoma β-cell line INS-1 was cultured as previously described (Zhou and Misler, 1996). The cells were maintained at 37°C in a humidified incubator supplemented with 5% CO_2_ and were subcultured twice per week. The cells were maintained in RPMI 1640 medium containing 11 mM D-glucose supplemented with 10% FBS (fetal bovine serum), 100 mg/ml penicillin-streptomycin, 10 mM HEPES, 1 mM sodium pyruvate, 2 mM L-glutamine, and 50 μM beta-mercaptoethanol (Invitrogen, Saint Aubin, France).

All experiments were performed at 37°C. INS-1 cells were plated on glass coverslips and incubated with bath solution containing the following (in mM): 136 NaCl, 4.2 KCl, 2.4 CaCl_2_, 1.2 KH_2_PO_4_, 1.2 MgSO_4_, 5 glucose, 10 HEPES, and 1 L-glutamine (pH 7.4). Individual coverslips were then placed in a metal chamber mounted on a heated stage. The INS-1 cells were stimulated with a solution containing the following (in mM): 70 NaCl, 70 KCl, 2.4 CaCl_2_, 1.2 KH_2_PO_4_, 1.2 MgSO_4_, 20 glucose, 10 HEPES, and 1 L-glutamine (pH 7.4) to trigger insulin granule exocytosis under the TIRF-SIM microscope.

Glucotoxicity conditions were obtained by exposing INS-1 cells to 20 mM and 30 mM glucose (high glucose) for 120 h. The control cells were INS-1 cells exposed to standard culture medium containing 11 mM glucose (Control) for 120 h. Each respective culture medium was changed after 48 h and replaced with the same culture medium until 120 h.

### Antibodies

The following primary antibodies were used for immunofluorescence and western blot analysis: polyclonal guinea pig anti-insulin antibody (1:200, Dako, Carpentaria, CA, United States); mouse monoclonal anti-syntaxin-1A (1:1000, SySy); mouse monoclonal anti-SNAP-25 (1:1000, SySy); rabbit monoclonal anti-VAMP2 (1:1000, Abcam); rabbit monoclonal anti-tubulin (1:1000, Abcam). The secondary antibody was DyLight 488 goat anti-guinea pig IgG (1:500, Thermo); IRDye 800CW goat anti-rabbit IgG (1:1000, LI-COR Biosciences); and IRDye 680CW goat anti-mouse IgG (1:1000, LI-COR Biosciences).

### ELISA Detection of Insulin Secretion

INS-1 cells cultured in control or high glucose conditions were washed with 1 ml of KRBB buffer (125 mM NaCl, 5.9 mM KCl, 2.56 mM CaCl_2_, 1.2 mM MgCl_2_, 1 mM L-glutamine, 25 mM HEPES, and 1 g/L BSA) containing 5 mM glucose, preincubated for 1 h at 37°C and then transferred to KRBB containing 16.7 mM glucose for 20 min at 37°C. The incubation solution was then collected, cell lysates were prepared by incubating for 30 min in RIPA buffer at 4°C, and the insulin levels were quantified using a rat/mouse insulin ELISA kit according to the manufacturer’s instructions (EZRMI-13K, Millipore). Insulin secretion was normalized to the total insulin content determined from the cell lysates, and the values shown in Supplementary Figure 1 were detected by normalizing 4 × 10^5^ cells.

### RNA Extraction and Real-Time Quantitative RT-PCR

Total RNA was extracted from INS-1 cells using the RNeasy Mini Kit (74104, QIAGEN). First-strand complementary DNA was synthesized from total RNA using TransScript One-Step gDNA Removal and cDNA Synthesis SuperMix (AT311-03, TransGenBiotech). Real-time PCR was performed on an Eppendorf RealPlex2 system using TransStart Top Green qPCR SuperMix (AQ131-03, TransGenBiotech). The qPCR primers used to detect *VAMP2*, *syntaxin-1A*, *and SNAP-25* expression were VAMP2-F (5′-GGGAGTCTGGACTTTTGGGG-3′), VAMP2-R (5′-GAAACGGGGTAAGGGAAG-3′), syntaxin-1A-F (5′-CATGGACTCCAGCATCTCGAA-3′), syntaxin-1A-R (5′-TCCATGAACATGTCGTGCAGC-3′), SNAP-25-F (5′-TCGGGAACCTCCGTCAC-3′), and SNAP-25-R (5′-AATTCTGGTTTTGTTGGAATCAG-3′). RNA transcript levels were quantified using the 2^–ΔΔCt^ method.

### Immunofluorescence

For immunofluorescence labeling, cell samples were fixed with 4% paraformaldehyde in PBS for 15 min, followed by permeabilization in PBS containing 0.5% Triton X-100 (MERCK, Billerica, MA, United States) for 10 min and blocking in PBS containing 5% bovine serum for 60 min. The samples were incubated for 60 min in PBS containing primary antibodies and 2.5% bovine serum and then exposed to fluorescent dye-conjugated secondary antibodies for 60 min at 37°C. The cell samples were scanned with an Olympus IX81 (Olympus, Tokyo, Japan).

Insulin granules of INS-1 cells were labeled with anti-insulin antibodies. Besides, we performed the Z-series analysis of the whole-cell and calculated the averaged insulin vesicle number by normalizing per cell size. The insulin granule density in Supplementary Figure 2 was defined as the number of insulin puncta per cell volume (number/μm^3^). All the Immunofluorescence images were shown as maximum intensity projection throughout the cell and were determined by ImageJ (National Institutes of Health^1^).

### Western Blot Assay

The cell samples were washed with PBS and homogenized on ice with lysate buffer [150 mM NaCl, 1% NP-40, 0.5% deoxycholic acid, 0.1% SDS, 50 mM Tris, pH 8.0, 1 mM PMSF, and 2% proteinase inhibitor (539134, Calbiochem)]. The homogenates were centrifuged at 16,000 *g* for 15 min at 4°C, and the centrifuged supernatant was adjusted to same total protein concentration following protein quantification by a standard BCA method. Proteins were electrophoresed and transferred to nitrocellulose filter membranes. Each membrane was blocked by incubation for 1 h with PBS containing 0.1% Tween-20 (v/v) and 5% non-fat dried milk (w/v). After washing with 0.1% Tween-20 containing PBS (PBST), the blots were incubated with primary antibodies at 4°C overnight in PBST containing 2% bovine serum albumin (BSA). Secondary antibodies were then applied at room temperature for 1 h. Blots were scanned with an Odyssey infrared imaging system (LI-COR Biosciences) and quantified with ImageJ. At least three independent western blots were conducted, and one typical blot is presented.

### Fluorescence Imaging

All confocal microscope images in Supplementary Figure 2 were generated using an inverted fluorescence microscope (Olympus, IX81) and a Laser Scanning Confocal system (Yokogawa, CSU-X1) with a 100×, 1.30 numerical aperture (NA) oil objective lens. The confocal settings used for image capture were held constant when samples were being compared. Images were quantified and analyzed using ImageJ software (National Institutes of Health).

TIRF-SIM imaging. The imaging system has been schematically illustrated (Huang et al., 2018). Briefly, it was based on a commercially available inverted fluorescence microscope (IX83, Olympus) equipped with a TIRF objective (Apo N 100×/1.7 HI Oil, Olympus), and images were captured by a sCMOS camera (Flash 4.0 V2, Hamamatsu, Japan). For TIRF-SIM, nine raw images are required to reconstruct one super-resolved image. Reaching a frame rate of 873 Hz, the temporal resolution for the non-overlapping reconstruction of SR images was 97 Hz. Because the three images in one orientation were independent of the images in the other two orientations, we proposed the reconstruction of time-lapse SIM images using overlapping raw image sequences (“rolling”) (Huang et al., 2018). By implementing this algorithm, we further increased the temporal resolvability to 291 Hz. All the images were obtained at a frame rate of 291 Hz except for Figure 2A and Supplementary Figure 2.

A 488-nm laser was used to excite GFP/pHluorin. To visualize VAMP2-pHluorin-labeled granule numbers (Figure 2A), the TIRF illumination was adjusted to 2D-SIM. Each pixel from the camera was 32.5 nm × 32.5 nm. The release probability in Figure 2E was defined to describe the chances of vesicles to fuse to the plasma membrane, which can be calculated as (number of fusion events) / (granule number per cell).

### Data Analysis and Statistics

Image processing was primarily performed using ImageJ software. The reconstruction of the images acquired by TIRF-SIM or 2D-SIM was accomplished using customized MATLAB software (2016a). The data analysis and curve fitting were performed using Igor Pro software (6.11, WaveMetrics, Lake Oswego, OR, United States) and GraphPad 7.0 software. To analyze different stages of vesicle fusion, we determined the time interval between the initiation of the fast rise in fluorescence intensity (the intersection of the stable linear fit at the bottom and the fitted fast rise) to the initiation of slow rise (the intersection of the fitted fast rise slope and the fitted slow rise) as the **t_1_**; the time interval between the initiation of the slow rise in fluorescence intensity to the time point of the initiation of stable fluorescence (the intersection of the fitted slow rise and the stable linear fit at the top) as the **t_2_**; the time interval between the initiation of stable fluorescence to the time point of the initiation of fluorescence decay (the intersection of the stable linear fit at the top and the fitted exponential decay) as the **t_3_**.

The results are presented as the mean ± SEM. Statistical significance was evaluated using Student’s *t*-test for single Gaussian-distributed datasets or the Mann–Whitney rank sum test for non-single Gaussian-distributed datasets. The asterisks ^∗^, ^∗∗^, and ^∗∗∗^ denote statistical significance at *p* < 0.05, *p* < 0.01, and *p* < 0.001, respectively.

## Data Availability Statement

All relevant data is contained within the article. The raw data supporting the conclusions of this article will be made available by the authors, without undue reservation.

## Author Contributions

LC and LW conceived and supervised the research. GY designed and performed the experiments, and data analysis and prepared the figures. GY and LW wrote the manuscript. All authors participated in discussions and data interpretation. All authors contributed to the article and approved the submitted version.

## Conflict of Interest

LC was employed by Shenzhen Bay Laboratory. The remaining authors declare that the research was conducted in the absence of any commercial or financial relationships that could be construed as a potential conflict of interest.

## Footnotes

1 https://imagej.nih.gov/ij/

## Supplementary Material

Supplementary Figure 1 Reduced glucose-stimulated insulin secretion in INS-1 cells cultured under hyperglycemic conditions. Insulin secretion of INS-1 cells under control (11 mM) and high glucose culture medium (20 and 30 mM glucose) (*n* = 4 repeats per condition). ^∗^*p* < 0.05, ^∗∗^*p* < 0.01, ^∗∗∗^*p* < 0.001.

Supplementary Figure 2 Hyperglycemia leads to the decreased insulin granule density in INS-1 cells. Immunofluorescence analysis of vesicles content (labeled with insulin antibody), showing **(A)** subcellular distribution and **(B)** granule density under normal glucose (11 mM, *n* = 20 cells) and supraphysiological glucose (20 mM: *n* = 25 cells; 30 mM: *n* = 18 cells). Scale bar: 5 μm. ^∗^*p* < 0.05, ^∗∗^*p* < 0.01, ^∗∗∗^*p* < 0.001.

Supplementary Figure 3 Unaltered vesicle diameters after long-term exposure to high glucose culture conditions. **(A–C)** We applied 2 min of 50 mM NH_4_Cl incubation to neutralize the acidic pH within the vesicles and then estimated the diameters of VAMP2-pHluorin vesicles in INS-1 cells cultured in control and high glucose conditions. **(A)** Control, 11 mM glucose; **(B)** 20 mM glucose; **(C)** 30 mM glucose (*n* = 120 vesicles per condition). ^∗^*p* < 0.05, ^∗∗^*p* < 0.01, ^∗∗∗^*p* < 0.001.

Supplementary Figure 4 Hyperglycemia decreases the expression of core SNARE complex in INS-1 cells. Quantification of mRNA expression **(A)** and protein expression of SNARE complex proteins **(B)** in INS-1cells. Values were normalized to intensities of control (11 mM glucose). ^∗^*p* < 0.05, ^∗∗^*p* < 0.01, ^∗∗∗^*p* < 0.001.

Supplementary Figure 5 Decreased SNARE proteins altered the fusion kinetics of dot fusion and kiss-and-stay events. **(A,B)** Dot fusion intermediates upon KD of syntaxin-1A **(A)**, SNAP-25 **(B)**, (Control: *n* = 40 fusion events from 8 cells; Stx-1A KD: *n* = 25 fusion events from 10 cells; SNAP-25 KD: *n* = 30 fusion events from 10 cells) and SNARE proteins KD+ Rescue (Stx-1A: *n* = 45 fusion events from 7 cells; SNAP-25: *n* = 30 fusion events from 6 cells). **(C)** Dynamics of kiss-and-stay events in INS-1 cells with KD of syntaxin-1A, SNAP-25 (Control: *n* = 10 fusion events from 5 cells; Stx-1A KD: *n* = 15 fusion events from 4 cells; SNAP-25 KD: *n* = 14 fusion events from 5 cells) and SNARE proteins KD+ Rescue (Stx-1A: *n* = 18 fusion events from 5 cells; SNAP-25: *n* = 15 fusion events from 4 cells). ^∗^*p* < 0.05, ^∗∗^*p* < 0.01, ^∗∗∗^*p* < 0.001.

## References

[B1] Albillos A. Dernick G. Horstmann H. Almers W. Alvarez, de Toledo G. (1997). The exocytotic event in chromaffin cells revealed by patch amperometry. *Nature* 389 509–512. 10.1038/39081 9333242

[B2] Alvarez, de Toledo G. Fernández-Chacón R. Fernández J. M. (1993). Release of secretory products during transient vesicle fusion. *Nature* 363 554–558. 10.1038/363554a0 8505984

[B3] Anantharam A. Onoa B. Edwards R. H. Holz R. W. Axelrod D. (2010). Localized topological changes of the plasma membrane upon exocytosis visualized by polarized TIRFM. *J. Cell Biol.* 188 415–428. 10.1083/jcb.200908010 20142424 PMC2819686

[B4] Bao H. Das D. Courtney N. A. Jiang Y. Briguglio J. S. Lou X. (2018). Dynamics and number of trans-SNARE complexes determine nascent fusion pore properties. *Nature* 554 260–263. 10.1038/nature25481 29420480 PMC5808578

[B5] Bendahmane M. Bohannon K. P. Bradberry M. M. Rao T. C. Schmidtke M. W. Abbineni P. S. (2018). The synaptotagmin C2B domain calcium-binding loops modulate the rate of fusion pore expansion. *Mol. Biol. Cell* 29 834–845. 10.1091/mbc.E17-11-0623 29444959 PMC5905296

[B6] Bensellam M. Laybutt D. R. Jonas J.-C. (2012). The molecular mechanisms of pancreatic β-cell glucotoxicity: recent findings and future research directions. *Mol. Cell. Endocrinol.* 364 1–27. 10.1016/j.mce.2012.08.003 22885162

[B7] Caromile L. A. Oganesian A. Coats S. A. Seifert R. A. Bowen-Pope D. F. (2010). The neurosecretory vesicle protein phogrin functions as a phosphatidylinositol phosphatase to regulate insulin secretion. *J. Biol. Chem.* 285 10487–10496. 10.1074/jbc.M109.066563 20097759 PMC2856256

[B8] Chow R. H. von Rüden L. Neher E. (1992). Delay in vesicle fusion revealed by electrochemical monitoring of single secretory events in adrenal chromaffin cells. *Nature* 356 60–63. 10.1038/356060a0 1538782

[B9] Collins S. C. Do H. W. Hastoy B. Hugill A. Adam J. Chibalina M. V. (2016). Increased expression of the diabetes gene SOX4 reduces insulin secretion by impaired fusion pore expansion. *Diabetes* 65 1952–1961. 10.2337/db15-1489 26993066 PMC4996324

[B10] Flatt P. R. Boquist L. Hellman B. (1980). Calcium and pancreatic β-cell function. The mechanism of insulin secretion studied with the aid of lanthanum. *Biochem. J.* 190 361–372. 10.1042/bj1900361 6162455 PMC1162101

[B11] Gaisano H. Y. Ostenson C.-G. Sheu L. Wheeler M. B. Efendic S. (2002). Abnormal expression of pancreatic islet exocytotic soluble N-ethylmaleimide-sensitive factor attachment protein receptors in Goto-Kakizaki rats is partially restored by phlorizin treatment and accentuated by high glucose treatment. *Endocrinology* 143 4218–4226. 10.1210/en.2002-220237 12399415

[B12] Guček A. Gandasi N. R. Omar-Hmeadi M. Bakke M. Døskeland S. O. Tengholm A. (2019). Fusion pore regulation by cAMP/Epac2 controls cargo release during insulin exocytosis. *eLife* 8:e41711. 10.7554/eLife.41711 31099751 PMC6557626

[B13] Hanna S. T. Pigeau G. M. Galvanovskis J. Clark A. Rorsman P. MacDonald P. E. (2009). Kiss-and-run exocytosis and fusion pores of secretory vesicles in human beta-cells. *Pflugers Arch. Eur. J. Physiol.* 457 1343–1350. 10.1007/s00424-008-0588-0 18795319

[B14] Huang X. Fan J. Li L. Liu H. Wu R. Wu Y. (2018). Fast, long-term, super-resolution imaging with Hessian structured illumination microscopy. *Nat. Biotechnol.* 36 451–459. 10.1038/nbt.4115 29644998

[B15] Kawahito S. Kitahata H. Oshita S. (2009). Problems associated with glucose toxicity: role of hyperglycemia-induced oxidative stress. *World J. Gastroenterol.* 15 4137–4142. 10.3748/wjg.15.4137 19725147 PMC2738809

[B16] Klec C. Ziomek G. Pichler M. Malli R. Graier W. F. (2019). Calcium signaling in ß-cell physiology and pathology: a revisit. *Int. J. Mol. Sci.* 20:6110. 10.3390/ijms20246110 31817135 PMC6940736

[B17] Lemaire K. Schuit F. (2012). Integrating insulin secretion and ER stress in pancreatic β-cells. *Nat. Cell Biol*. 14 979–981. 10.1038/ncb2594 23033046

[B18] Ma L. Bindokas V. P. Kuznetsov A. Rhodes C. Hays L. Edwardson J. M. (2004). Direct imaging shows that insulin granule exocytosis occurs by complete vesicle fusion. *Proc. Natl. Acad. Sci. U S A.* 101 9266–9271. 10.1073/pnas.0403201101 15197259 PMC438965

[B19] MacDonald P. E. Braun M. Galvanovskis J. Rorsman P. (2006). Release of small transmitters through kiss-and-run fusion pores in rat pancreatic beta cells. *Cell Metab.* 4 283–290. 10.1016/j.cmet.2006.08.011 17011501

[B20] Miesenböck G. De Angelis D. A. Rothman J. E. (1998). Visualizing secretion and synaptic transmission with pH-sensitive green fluorescent proteins. *Nature* 394 192–195. 10.1038/28190 9671304

[B21] Nagamatsu S. Nakamichi Y. Yamamura C. Matsushima S. Watanabe T. Ozawa S. (1999). Decreased expression of t-SNARE, syntaxin 1, and SNAP-25 in pancreatic beta-cells is involved in impaired insulin secretion from diabetic GK rat islets: restoration of decreased t-SNARE proteins improves impaired insulin secretion. *Diabetes* 48 2367–2373. 10.2337/diabetes.48.12.2367 10580425

[B22] Ornberg R. L. Reese T. S. (1981). Beginning of exocytosis captured by rapid-freezing of Limulus amebocytes. *J. Cell Biol.* 90 40–54. 10.1083/jcb.90.1.40 7195907 PMC2111820

[B23] Ostenson C.-G. Efendic S. (2007). Islet gene expression and function in type 2 diabetes; studies in the Goto-Kakizaki rat and humans. *Diabetes Obesity Metab.* 9(Suppl. 2), 180–186. 10.1111/j.1463-1326.2007.00787.x 17919192

[B24] Rizo J. Südhof T. C. (2002). Snares and Munc18 in synaptic vesicle fusion. *Nat. Rev. Neurosci.* 3 641–653. 10.1038/nrn898 12154365

[B25] Rutter G. A. Loder M. K. Ravier M. A. (2006). Rapid three-dimensional imaging of individual insulin release events by Nipkow disc confocal microscopy. *Biochem. Soc. Trans.* 34(Pt 5), 675–678. 10.1042/BST0340675 17052172

[B26] Shi L. Shen Q.-T. Kiel A. Wang J. Wang H.-W. Melia T. J. (2012). SNARE proteins: one to fuse and three to keep the nascent fusion pore open. *Science* 335 1355–1359. 10.1126/science.1214984 22422984 PMC3736847

[B27] Shin W. Ge L. Arpino G. Villarreal S. A. Hamid E. Liu H. (2018). Visualization of membrane pore in live cells reveals a dynamic-pore theory governing fusion and endocytosis. *Cell* 173 934–945.e12. 10.1016/j.cell.2018.02.062 29606354 PMC5935532

[B28] Sudhof T. C. (2004). The synaptic vesicle cycle. *Ann. Rev. Neurosci.* 27 509–547. 10.1146/annurev.neuro.26.041002.131412 15217342

[B29] Takahashi N. Kishimoto T. Nemoto T. Kadowaki T. Kasai H. (2002). Fusion pore dynamics and insulin granule exocytosis in the pancreatic islet. *Science* 297 1349–1352. 10.1126/science.1073806 12193788

[B30] Tomita T. (2016). Apoptosis in pancreatic β-islet cells in Type 2 diabetes. *Bosnian J. Basic Med. Sci.* 16 162–179. 10.17305/bjbms.2016.919 27209071 PMC4978108

[B31] Tsuboi T. Rutter G. A. (2003). Multiple forms of “kiss-and-run” exocytosis revealed by evanescent wave microscopy. *Curr. Biol. CB* 13 563–567. 10.1016/s0960-9822(03)00176-3 12676086

[B32] Unger R. H. Grundy S. (1985). Hyperglycaemia as an inducer as well as a consequence of impaired islet cell function and insulin resistance: implications for the management of diabetes. *Diabetologia* 28 119–121. 10.1007/BF00273856 3888754

[B33] Weber T. Zemelman B. V. McNew J. A. Westermann B. Gmachl M. Parlati F. (1998). SNAREpins: minimal machinery for membrane fusion. *Cell* 92 759–772. 10.1016/s0092-8674(00)81404-x 9529252

[B34] Wightman R. M. Jankowski J. A. Kennedy R. T. Kawagoe K. T. Schroeder T. J. Leszczyszyn D. J. (1991). Temporally resolved catecholamine spikes correspond to single vesicle release from individual chromaffin cells. *Proc. Natl. Acad. Sci. U S A.* 88 10754–10758. 10.1073/pnas.88.23.10754 1961743 PMC53009

[B35] Xie L. Zhu D. Dolai S. Liang T. Qin T. Kang Y. (2015). Syntaxin-4 mediates exocytosis of pre-docked and newcomer insulin granules underlying biphasic glucose-stimulated insulin secretion in human pancreatic beta cells. *Diabetologia* 58 1250–1259. 10.1007/s00125-015-3545-4 25762204

[B36] Zhang W. Khan A. Ostenson C.-G. Berggren P.-O. Efendic S. Meister B. (2002). Down-regulated expression of exocytotic proteins in pancreatic islets of diabetic GK rats. *Biochem. Biophys. Res. Commun.* 291 1038–1044. 10.1006/bbrc.2002.6555 11866470

[B37] Zhao Y. Fang Q. Herbst A. D. Berberian K. N. Almers W. Lindau M. (2013). Rapid structural change in synaptosomal-associated protein 25 (SNAP25) precedes the fusion of single vesicles with the plasma membrane in live chromaffin cells. *Proc. Natl. Acad. Sci. U S A.* 110 14249–14254. 10.1073/pnas.1306699110 23940346 PMC3761627

[B38] Zhou Z. Misler S. (1996). Amperometric detection of quantal secretion from patch-clamped rat pancreatic beta-cells. *J. Biol. Chem.* 271 270–277. 10.1074/jbc.271.1.270 8550572

[B39] Zhou Z. Misler S. Chow R. H. (1996). Rapid fluctuations in transmitter release from single vesicles in bovine adrenal chromaffin cells. *Biophys. J.* 70 1543–1552. 10.1016/S0006-3495(96)79718-7 8785312 PMC1225082

[B40] Zhu D. Koo E. Kwan E. Kang Y. Park S. Xie H. (2013). Syntaxin-3 regulates newcomer insulin granule exocytosis and compound fusion in pancreatic beta cells. *Diabetologia* 56 359–369. 10.1007/s00125-012-2757-0 23132338

