# Supplementary figures and images for: Hyperglycemia-Induced Dysregulated Fusion Intermediates in Insulin-Secreting Cells Visualized by Super-Resolution Microscopy
Source: Front Cell Dev Biol. 2021 Apr 15;9:650167. doi: 10.3389/fcell.2021.650167 (PMC8083903; doi:10.3389/fcell.2021.650167)

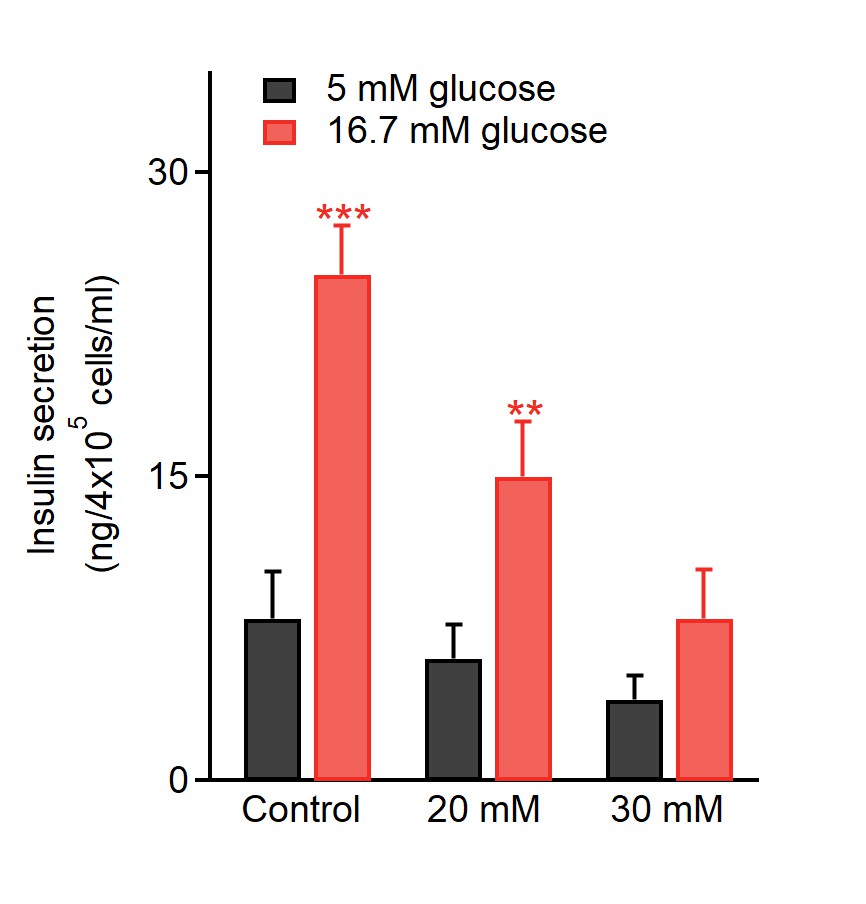

Supplement: Supplementary Figure 1 — Reduced glucose-stimulated insulin secretion in INS-1 cells cultured under hyperglycemic conditions. Insulin secretion of INS-1 cells under control (11 mM) and high glucose culture medium (20 and 30 mM glucose) (n = 4 repeats per condition). ∗p < 0.05, ∗∗p < 0.01, ∗∗∗p < 0.001. [file Image_1.JPEG]

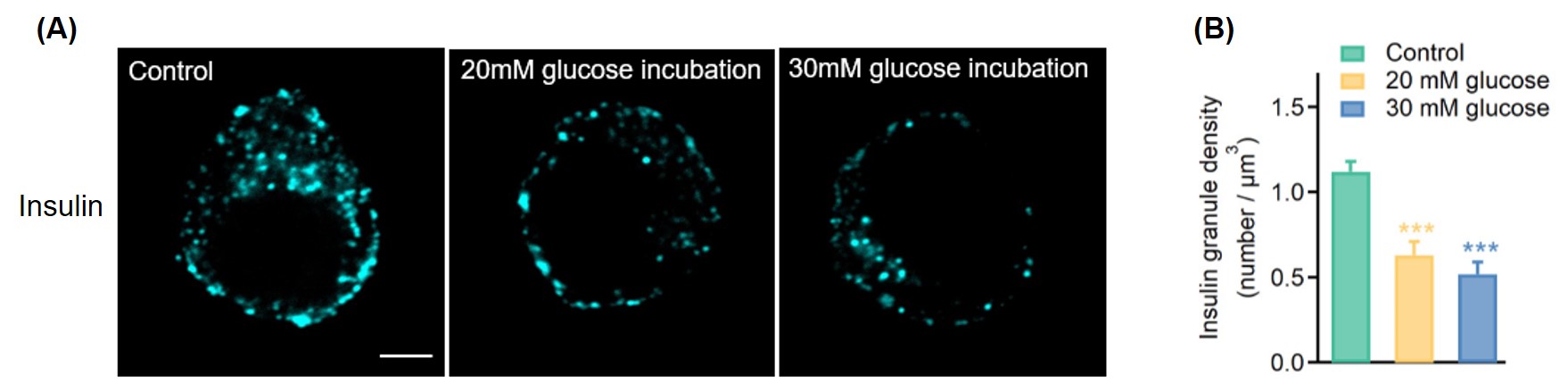

Supplement: Supplementary Figure 2 — Hyperglycemia leads to the decreased insulin granule density in INS-1 cells. Immunofluorescence analysis of vesicles content (labeled with insulin antibody), showing (A) subcellular distribution and (B) granule density under normal glucose (11 mM, n = 20 cells) and supraphysiological glucose (20 mM: n = 25 cells; 30 mM: n = 18 cells). Scale bar: 5 μm. ∗p < 0.05, ∗∗p < 0.01, ∗∗∗p < 0.001. [file Image_2.JPEG]

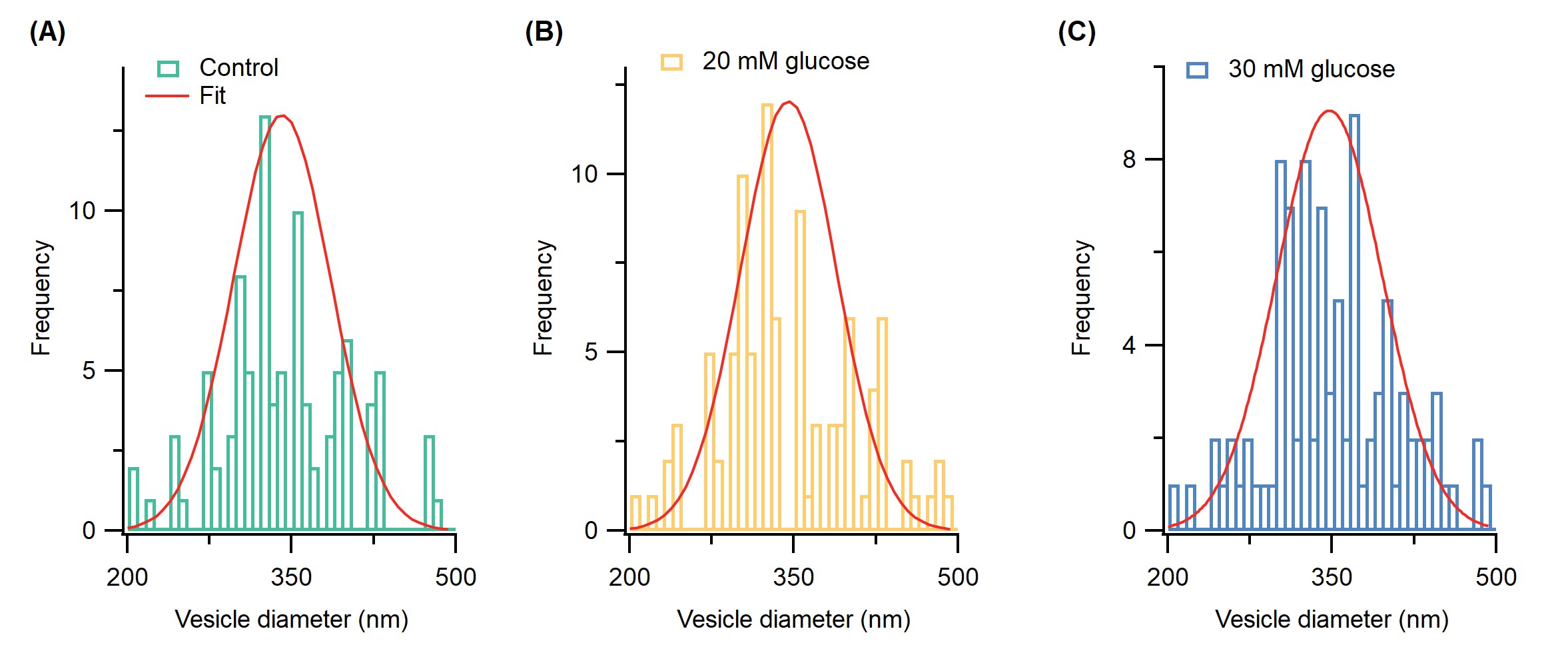

Supplement: Supplementary Figure 3 — Unaltered vesicle diameters after long-term exposure to high glucose culture conditions. (A–C) We applied 2 min of 50 mM NH4Cl incubation to neutralize the acidic pH within the vesicles and then estimated the diameters of VAMP2-pHluorin vesicles in INS-1 cells cultured in control and high glucose conditions. (A) Control, 11 mM glucose; (B) 20 mM glucose; (C) 30 mM glucose (n = 120 vesicles per condition). ∗p < 0.05, ∗∗p < 0.01, ∗∗∗p < 0.001. [file Image_3.JPEG]

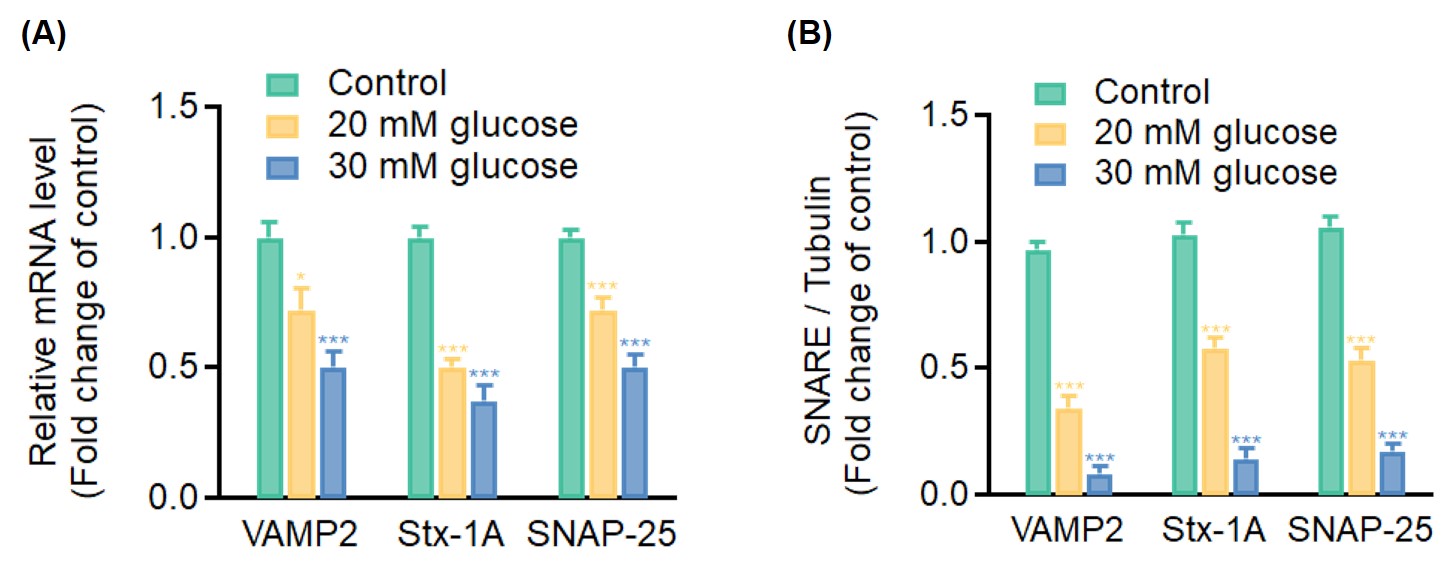

Supplement: Supplementary Figure 4 — Hyperglycemia decreases the expression of core SNARE complex in INS-1 cells. Quantification of mRNA expression (A) and protein expression of SNARE complex proteins (B) in INS-1cells. Values were normalized to intensities of control (11 mM glucose). ∗p < 0.05, ∗∗p < 0.01, ∗∗∗p < 0.001. [file Image_4.JPEG]

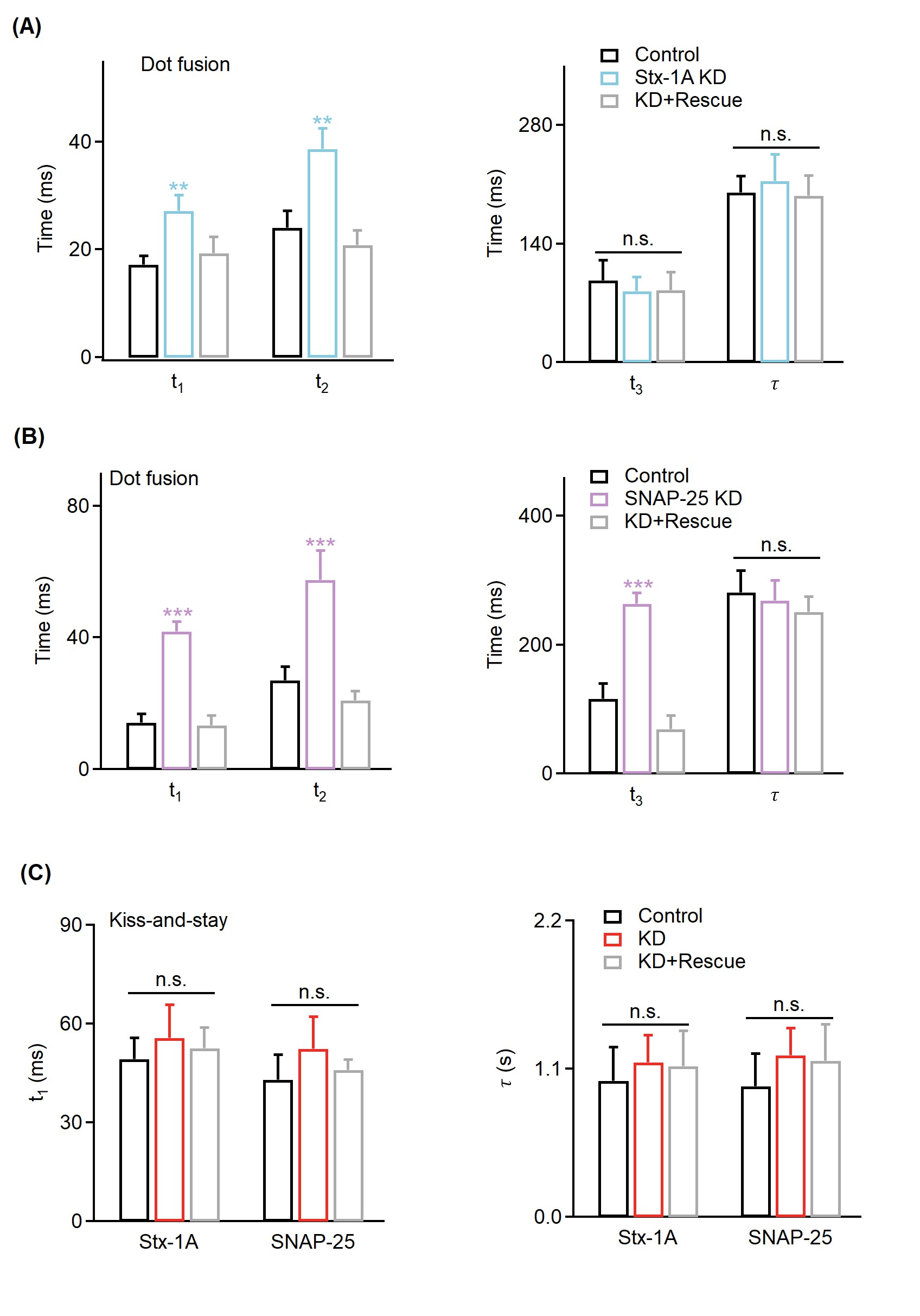

Supplement: Supplementary Figure 5 — Decreased SNARE proteins altered the fusion kinetics of dot fusion and kiss-and-stay events. (A,B) Dot fusion intermediates upon KD of syntaxin-1A (A), SNAP-25 (B), (Control: n = 40 fusion events from 8 cells; Stx-1A KD: n = 25 fusion events from 10 cells; SNAP-25 KD: n = 30 fusion events from 10 cells) and SNARE proteins KD+ Rescue (Stx-1A: n = 45 fusion events from 7 cells; SNAP-25: n = 30 fusion events from 6 cells). (C) Dynamics of kiss-and-stay events in INS-1 cells with KD of syntaxin-1A, SNAP-25 (Control: n = 10 fusion events from 5 cells; Stx-1A KD: n = 15 fusion events from 4 cells; SNAP-25 KD: n = 14 fusion events from 5 cells) and SNARE proteins KD+ Rescue (Stx-1A: n = 18 fusion events from 5 cells; SNAP-25: n = 15 fusion events from 4 cells). ∗p < 0.05, ∗∗p < 0.01, ∗∗∗p < 0.001. [file Image_5.JPEG]
